# Transthoracic Echocardiography in the Preoperative Assessment of Newborn Coarctation: Limiting Risks Associated with Advanced Imaging Techniques

**DOI:** 10.1097/pq9.0000000000000682

**Published:** 2023-09-28

**Authors:** Christina L. Benjamin, Erik G. Ellsworth, Roosevelt Bryant, Deepti P. Bhat

**Affiliations:** From the Center for Heart Care, Phoenix Children’s Hospital, Phoenix, Ariz.

## Abstract

A newborn male child with prenatally identified aortic arch hypoplasia presented to our facility for cardiac management. He had been started on prostaglandins at the delivery facility and was subsequently placed on a high-flow nasal cannula due to associated apnea. On the day of life three, the patient underwent cardiac computed tomography scan for delineation of his anatomy. The patient remained intubated after his imaging study in anticipation of surgical intervention, which took place at the age of 5 days. The patient required a peritoneal dialysis catheter placement 2 days after his procedure due to oliguria. He progressed into renal failure requiring continuous renal replacement therapy.

This patient was subsequently discussed at our departmental morbidity and mortality conference. The short time frame between contrast administration for the computed tomography and surgical intervention was thought to have contributed to his renal failure. We discussed the adequacy of transverse aortic arch imaging by echocardiogram and the utility of advanced imaging in the fragile neonatal period. This discussion resulted in our department asking, “Is transthoracic echocardiography accurate when diagnosing and characterizing aortic coarctation at our institution? Are advanced imaging studies necessary in instances of simple coarctation?”

## INTRODUCTION

Coarctation of the aorta (CoA) is a frequently diagnosed congenital heart defect, and surgical repair is the second most common cardiac intervention performed in the neonatal period.^1,2^ Transthoracic echocardiography (TTE) is the standard diagnostic modality for pediatric coarctation. However, advanced cross-sectional imaging, including cardiac computed tomography (CT) and magnetic resonance imaging (CMR), has been increasingly used for surgical planning to provide conclusive anatomical details.^3^

Although these studies provide detailed anatomic information, advanced imaging exposes children to anesthesia, intubation, and radiation risks. TTE is known to be a safe, noninvasive imaging modality in children, but limited data are available correlating TTE findings related to CoA with advanced imaging in these patients.

We investigated the current practice at our institute to evaluate and implement changes to reduce preoperative morbidity. The project aimed to determine our institution’s accuracy in the preoperative diagnosis and characterization of simple pediatric coarctation via the noninvasive modality of TTE in comparison to advanced imaging modalities. Given the morbidity associated with advanced imaging studies in the pediatric congenital heart disease population, we aimed to promote practice change that would limit the utilization of these imaging studies in routine cases.

## METHODS

A single-center retrospective chart review from January 2015 to December 2020 identified patients diagnosed with simple CoA by TTE. Simple CoA was defined as coarctation of the aorta without other significant intracardiac abnormalities, except for minor lesions such as a bicuspid aortic valve or small ventricular or atrial septal defects. In contrast, we defined a complex CoA in the presence of other significant lesions, such as hypoplastic left heart, vascular ring, and interrupted aortic arch. These exclusions were performed to identify those patients who underwent advanced imaging strictly evaluating their CoA, as our underlying question pertained to the over-use of advanced imaging techniques in this population. Therefore, we included all simple CoA patients with preoperative TTE and advanced imaging in the study.

We collected patient demographics and diagnostic data from all imaging modalities. The aorta was measured at specific anatomic locations for comparison: ascending aorta diameter via parasternal long axis views, proximal transverse arch diameter at the take-off of the brachiocephalic artery, distal transverse arch diameter before the left subclavian artery, and isthmus measurement in long segment CoA or narrowest diameter in discrete CoA. We obtained two sets of measurements and averaged from both studies to limit interoperative variability. We compared aortic arch findings and the accuracy of TTE with cardiac CT/CMR, using the Student paired *t* test. REDCap software was used for data management.

*Intervention*: We presented the study results at a departmental research and quality improvement meeting attended by the medical and surgical staff. Recommendations were made regarding a change in practice. In addition, we discussed barriers to the changes. This educational intervention’s impact on changing our institute’s practice will be studied after 5 years.

## RESULTS

Baseline characteristics are presented in Table 1. Of the 74 patients in the study, 33 patients were diagnosed with a discrete CoA (median age at diagnosis = 61 months), and 41 were diagnosed with long segment CoA (median age at diagnosis = 4 days). The average time from TTE to undergoing advanced imaging study was 11 days.

**Table 1.** Baseline Characteristics

Baseline Characteristics (N = 74)	
Gender	
Masculine	50 (67.7%)
Age at diagnosis	
Prenatal diagnosis	21 (28.4%)
Postnatal diagnosis (before the age of 1 year)	47 (63.5%)
Postnatal (after the age of 1 year)	24 (36.5%)
Coarctation type by TTE	
Discrete	33 (44.6%)
Long segment	41 (55.4%)
Pre-intervention advanced imaging	
Cardiac CT	54 (73%)
CMR	20 (27%)

Table 2 shows the anatomic determinants of CoA important to surgical planning, specifically for selecting the surgical approach (median sternotomy versus lateral thoracotomy). The table demonstrates a high level of agreement between the TTE findings and those of the advanced imaging study performed. Only one patient had a change in the CoA classification from long segment by TTE to discrete by cardiac CT. In 71 patients, aortic arch-sidedness and branching were accurately identified by TTE (96%). However, TTE did not visualize arch-sidedness well in two of the patients. In addition, the presence of an aberrant subclavian artery origin was missed in one patient, and branching was not well defined for two patients by TTE.

**Table 2.** Diagnostic Accuracy of TTE Compared with Cardiac CT/CMR

Diagnostic Accuracy of TTE Compared with Cardiac CT/CMR		
	No.	Agreement
Discrete versus long segment	73/74	99%
Aortic arch-sidedness	71/74	96%
Aortic arch branching	71/74	96%

We obtained discrete measurements of the aortic arch by both TTE and cardiac CT/CMR. Although the exact measurements of the segments of aorta segments differed between two-dimensional TTE and cross-sectional imaging (cardiac CT/CMR), there was no significant difference in the transverse arch, typically used to determine the surgical approach for CoA repair. A paired *t* test demonstrated a TTE mean measurement of 10.7 mm (SEM 0.8 mm) versus an advanced imaging measurement of 10.6 mm (SEM 0.7 mm) (*P =* 0.8). We noted that TTE measurements at the coarctation site tended to be smaller compared with cardiac CT/CMR, with measurements of 4.9 mm (SEM 0.3 mm) versus 5.8 mm (SEM 0.5 mm) (*P =* 0.02) in patients with discrete CoA. However, we did not consider this a clinically significant determinant for surgical planning.

## DISCUSSION

TTE is the most frequently used imaging modality in pediatric cardiology, though using advanced imaging modalities has become more routine. Unfortunately, advanced imaging studies in this young patient population increases preoperative morbidity. Also, anesthesia and intubation, typically required for CMR in children, heighten the risk of associated adverse events. These risks are even more pronounced in the congenital heart disease population. Adverse events, ranging from bronchospasm and mild hypoxia to significant pulmonary edema and hypotension, may occur in as many as 10.4% of pediatric patients with congenital heart disease.^4^

The risks associated with anesthesia are not only applicable in CMR but also in the neonatal population requiring cardiac CT, as many of these patients will require sedation and intubation for image optimization given their young age. The National Cancer Institute also recognizes that no amount of radiation is safe. Pediatric patients are more sensitive to radiation than adults, and with a presumed longer lifespan, children have more opportunities to display the negative consequences of radiation.^5^ One retrospective cohort study by Pearce et al demonstrated that cumulative radiation doses from CT scans in pediatric patients are associated with a heightened risk of leukemia and brain tumors.^6^ The utilization of cardiac CT/CMR exposes patients to increased risk compared with TTE but without a clear benefit in determining the surgical approach required in CoA repair.^1,7^

Previous studies have attempted to determine the predictive value of aortic arch measurements in the preoperative diagnosis and management of obstructive aortic arch lesions. These studies have demonstrated a wide variability in the use of preoperative diameter definitions and indexed values for hypoplastic arches. The variability of measurement locations does not lend well to a normative predictive value.^8^

Our quality improvement analysis shows that TTE had a high degree of accuracy in ascertaining arch-sidedness, branching pattern, and coarctation type. As most indications for surgical intervention on the arch relate to clinical findings (ventricular dysfunction, Doppler pressure gradients, diminished femoral pulses, upper extremity to lower extremity blood pressure discrepancy, etc) and not discrete measurements by imaging studies, we can infer that arch characteristics and coarctation type are most helpful in determining the specific surgical approach. Therefore, limiting the use of advanced imaging studies that expose pediatric patients to increased risk should be emphasized.

Limitations potentially influencing this study include the retrospective nature. When a TTE study had not appropriately evaluated a characteristic of the aortic arch, it was classified as inaccurate compared with advanced imaging, as all characteristics should be defined before surgical intervention in this population. In addition, we excluded patients with coarctation associated with other complex congenital heart disease (such as large VSD, single ventricle disease, and multilevel left ventricular obstructive lesions) to limit sampling bias. These patients commonly undergo advanced imaging studies for reasons outside coarctation characterization and, therefore, would not be influenced by the outcomes/recommendations of our investigation. Our inclusion and exclusion criteria also helped make a more homogenous population. As in many pediatric studies, the population available for investigation remained quite limited, limiting our sample size for this study.

## CONCLUSIONS

Our study demonstrated that the noninvasive modality of TTE is highly accurate for the preoperative assessment of simple CoA and anatomic definitions in most pediatric patients. Therefore, with its associated risks, advanced imaging may be reserved for special circumstances. After 5 years, we will reassess our institutional practices relating to the utilization of advanced cross-sectional imaging in this population.

## DISCLOSURE

The authors have no financial interest to declare in relation to the content of this article.
